# Multiple concurrent collagenase clostridium histolyticum injections to dupuytren’s cords: an exploratory study

**DOI:** 10.1186/1471-2474-13-61

**Published:** 2012-04-27

**Authors:** Stephen Coleman, David Gilpin, James Tursi, Greg Kaufman, Nigel Jones, Brian Cohen

**Affiliations:** 1 Brisbane Hand and Upper Limb Clinic, Level 9, 259 Wickham Terrace, Brisbane, Queensland, 4000, Australia; 2 Auxilium Pharmaceuticals, Inc, 40 Valley Stream Parkway, Malvern, PA, 19355, USA; 3 Auxilium Pharmaceuticals, Inc, Orchard Lea, Winkfield Lane, Windsor, SL4 4RU, UK

**Keywords:** Dupuytren’s disease, contracture, palpable cord, nonsurgical treatment, collagenase clostridium histolyticum (CCH), multiple injections, metacarpophalangeal joint, proximal interphalangeal joint

## Abstract

**Background:**

Dupuytren’s contracture (DC) is a progressive fibroproliferative disorder characterized by development of nodules and collagen cords within the palmar fascia of the hand. Collagenase clostridium histolyticum (CCH) is currently approved in adults with DC for the nonsurgical treatment of a single palpable cord during a 30-day treatment cycle. This open-label pilot study was designed to examine the safety, efficacy, and multiple-dose pharmacokinetics of injecting two cords (affected joints) with multiple doses of CCH concurrently into the same hand in subjects with DC and multiple contractures.

**Methods:**

Twelve subjects with DC were enrolled, each with ≥3 contractures caused by palpable cords. Efficacy assessments were taken 30 days after treatment and adverse events (AEs) were recorded throughout. In the first treatment period, all subjects were injected with a single dose of CCH (0.58 mg) into a single cord. The same subjects entered a second treatment period 30 days later, where two different cords (affected joints) were injected concurrently on the same hand. A finger extension procedure was performed 24 hours after each administration of CCH to disrupt the enzymatically weakened cord.

**Results:**

For metacarpophalangeal (MP) joints, mean contracture reduction per joint treated was 29.0 ± 20.7 degrees following single injection vs 30.3 ± 10.9 degrees per treated joint following multiple injections. For proximal interphalangeal (PIP) joints, mean reduction in contracture was 30.7 ± 21.1 and 22.1 ± 4.9 degrees per treated joint, respectively, for the two periods. All patients (100%) were either “quite satisfied” or “very satisfied” following either treatment cycle. The most common treatment-related AEs were edema peripheral, contusion, and pain in the treated extremity; the differences in severity for local effects of the injections were minimal between treatment periods. No serious treatment-related AEs or systemic complications were reported.

**Conclusion:**

These results provide preliminary evidence that two cords (affected joints) can be treated concurrently with CCH with similar efficacy and safety as cords treated individually in a sequential fashion. Multiple concurrent injections would eliminate the 30-day wait between single treatments and allow for rapid and effective treatment of patients with multiple affected joints, a significant advantage for both patient and physician.

**Trial registration:**

Australian New Zealand Clinical Trials Registry #ACTRN12610001045000.

## Background

Dupuytren’s contracture (DC) is a chronic, progressive fibroproliferative disorder that affects the hand. In the early stages of DC, abnormal myofibroblasts contribute to the formation of nodules, and as the disease progresses collagen deposits in the palm lead to the development of pathologic cords. Subsequent contraction of these cords results in flexion deformity and ultimately in contracture of the metacarpophalangeal (MP) and proximal interphalangeal (PIP) joints [1,2]. MP and PIP joints of the ring and small finger are most commonly affected [3,4]. Bilateral disease occurs frequently, and is considered to be a risk factor for development of an aggressive course of disease and a higher incidence of disease recurrence following surgical correction [4,5].

There is currently no cure for DC, and surgical correction of contractures has typically been the only accepted treatment option once the disease has progressed to the point of limiting the patient’s daily activities. Generally, surgical intervention is indicated once the contracture has increased to >30 degrees for MP joints and >15 degrees for PIP joints with identification of a palpable cord [1,6]. Several different surgical procedures are available for correction of DC, including simple fasciotomy, needle aponeurotomy, limited, partial, or regional fasciectomy, total or radical fasciectomy, and dermofasciectomy [1,7,8]. The different surgical procedures can address multiple affected joints on the same hand and generally have good results, but there can be complications from surgery, such as nerve or artery injury [9,10]. Particularly, the length of recovery time needed to return to daily activities following surgery is of concern to many patients [6].

Collagenase clostridium histolyticum (CCH; Xiaflex®, Xiapex®; Auxilium Pharmaceuticals/Pfizer Inc.) is a clinically effective, minimally invasive nonsurgical treatment of DC with a palpable cord that may have a better morbidity profile than surgical interventions [11,12]. CCH is comprised of a fixed-ratio mixture of two purified enzymes, a clostridial type I collagenase (AUX-I) and a clostridial type II collagenase (AUX-II), that work synergistically to enzymatically disrupt the collagen structure within DC cords [11]. Previously published phase III clinical studies have demonstrated CCH to be a safe and effective treatment option for patients with DC [13,14]. In both studies, more patients treated with CCH had a reduction in contracture to 0–5 degrees compared to placebo (64.0% vs 6.8% and 44.4% vs 4.8%, respectively; p < 0.001 for each study). The vast majority of AEs were local, mild to moderate in severity, confined to the treated extremity, and generally resolved without intervention prior to the next injection [13,14]. CCH is indicated for injection of only one cord at a time, and any additional palpable cords with contractures of MP or PIP joints should be injected with CCH sequentially with approximately 4 weeks between injections. The current study was conducted to determine if concurrent administration of two 0.58-mg injections of CCH into two palpable cords on the same hand in subjects with DC would result in comparable safety and efficacy results as to a single injection into one cord.

## Methods

### Study population

This open-label study was conducted at a single center in Australia. Subjects were eligible for study inclusion if they were in general good health and between the ages of 18 and 70. Subjects were required to have a diagnosis of DC with at least 3 fixed-flexion contractures, caused by palpable cords, that were ≥20  in PIP and/or MP joints in fingers (not the thumbs). The disease had to be severe enough that the subjects could not simultaneously place the affected finger(s) and palm flat against a table top. Exclusion criteria included previous treatment of the selected joints within 90 days of first dose of study drug; chronic muscular, neurologic, or neuromuscular disorders affecting the hands; known allergy to collagenase; collagenase treatment or treatment with any investigational drug within 30 days of first dose of study drug; anticoagulant within 7 days of first dose of study drug (with the exception of low-dose aspirin or NSAIDs); breastfeeding or pregnancy; and known history of stroke, bleeding disorder, or other medical condition that in the opinion of the investigator would compromise the subjects’ safety or the study objectives. All participants provided written informed consent and were free to discontinue at any time. The study protocol was approval by a local ethics committee (Queensland Institute of Medical Research, HREC #P1315), and research was carried out in compliance with the Declaration of Helsinki as currently amended.

### Study design

**Figure 1:**
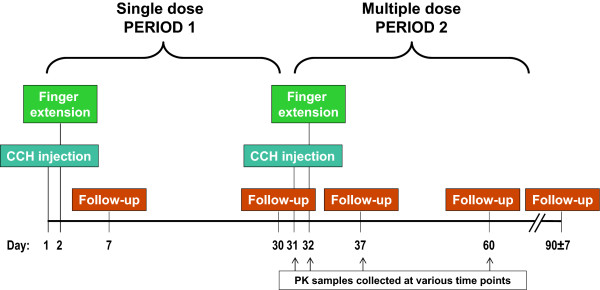
**Study design.** CCH = collagenase clostridium histolyticum; PK = pharmacokinetic.

An overview of the study design can be found in Figure 1. In the first treatment period, all subjects were given a single injection of CCH (0.58 mg; volume of 0.25 mL for MP joints and 0.20 mL for PIP joints) into a single cord. A finger extension procedure was conducted within 24 hours post-dosing, as described previously [14]. Local anesthesia could be used during the finger extension procedure in the first treatment period, but was not required. Subjects were bandaged for the first few days following the finger extension procedure and then were fitted for a splint to be worn each night for up to 4 months.

The same subjects entered a second treatment period 30 days later, where two different affected joints on the same hand were treated during the same visit (ie, patients received two concurrent 0.58-mg CCH doses). Collection of blood samples for pharmacokinetic analysis began on day 31, on the same visit as the injection for treatment period 2. The finger extension procedure without local anesthesia (to avoid any possible confounding effects on the pharmacokinetic evaluation) was performed at approximately 24 hours.

### Efficacy and safety assessments

Efficacy assessments, including joint contracture (measured by finger goniometry) and range of motion of the affected joint were made before dosing (day 1 for treatment period 1; day 31 for treatment period 2), and at follow-up visits during each treatment period (days 7 and 30 for treatment period 1; days 37 and 60 for treatment period 2). Subject satisfaction with treatment was reported at the end of each treatment period (day 30 and day 60) using a five-level Likert response scale (1. Very Satisfied, 2. Quite Satisfied, 3. Neither Satisfied nor Dissatisfied, 4. Quite Dissatisfied, 5. Very Dissatisfied).

Safety was monitored by recording AEs (relationship to drug assessed as probable, possible or not related), vital sign measurements, laboratory measurements, and specific local treatment effects, including bruising, swelling, pain, lymphadenopathy, and pruritus. Assessment of the severity of local treatment effects was made using a 6-point scale from none (0) to extensive (5). Blood samples for the determination of plasma anti-AUX-I and anti-AUX-II antibody levels were collected (days 1, 30, 60). Blood samples for pharmacokinetic evaluation were taken 15 minutes pre-dose on day 31 and at the following time points following the multiple dose administration: 5, 10, 20, and 30 minutes and 1, 2, 4, 8, 12, and 24 hours (immediately following the finger extension procedure); also, day 37 and 60.

### Statistical analyses

The safety population included all enrolled subjects. All subjects were also included in the pharmacokinetic population that received multiple injections of CCH in treatment period 2. Efficacy, safety, and pharmacokinetic variables are summarized with descriptive statistics.

## Results

### Patient characteristics

Among the 12 subjects (11 males, 1 female), the mean age was 64 ± 5.5 years and the mean age at symptom onset was 50.5 ± 11.1 years. Six patients had a family history of DC and 5 had previously undergone surgical correction (2 fasciotomy, 3 fasciectomy), but the other 7 patients had not received any treatment for DC. Of the patients who had previously undergone surgical correction, only one was injected with CCH in the same joint that had prior surgical correction, and one other patient had surgery (MP joint) and a CCH injection (PIP joint) in the same finger but in two other joints. Additional baseline patient information can be found in Table 1. Over the course of the study, 22 MP joints and 14 PIP joints were treated (Table 2). In treatment period 1, 5 MP joints and 7 PIP joints were treated. In treatment period 2, 17 MP joints and 7 PIP joints were treated. Most patients (n = 8) had joints treated on different hands for treatment period 1 and 2, while 4 patients had joints treated on the same hand but on different fingers. In treatment period 2, 5 subjects had an MP and a PIP joint treated on the same finger, 2 subjects had an MP and a PIP joint treated on different fingers, and 5 subjects had two MP joints treated on different fingers.

**Table 1:** Baseline patient characteristics (n = 12)

**Characteristic**	**Total**
Mean age, years (SD)	63.7 (5.5)
Male, n (%)	11 (91.7)
Ethnicity, n (%)^a^	
White, non-Hispanic	12 (100.0)
Weight (kg)	
Mean (SD)	87.7 (8.4)
Range	74.0, 100.0
Height (cm)	
Mean (SD)	177.2 (4.9)
Range	170.0, 188.0
Family history of Dupuytren’s contracture, n (%)	6 (50.0)
Age at symptom onset, years	
Mean (SD)	50.5 (11.1)
Range	23.0, 65.0
Prior treatment for Dupuytren’s contracture, n (%)	
None	7 (58.3)
Fasciotomy	2 (16.7)
Fasciectomy	3 (25.0)
Time since last hand surgery, years (n = 5)	
Mean (SD)	5.6 (4.9)
Range	0.3, 10.0

^a^Race or ethnic group was self-reported.

**Table 2:** Baseline joint characteristics for treatment periods 1 and 2

**Characteristic**	**Joints in Period 1 (n = 12)**	**Joints in Period 2 (n = 24)**
**MP joints, n (%)**	**5 (41.7)**	**17 (70.8)**
Low baseline severity (≤50°)	5 (41.7)	15 (62.5)
High baseline severity (>50°)	0 (0.0)	2 (8.3)
Little finger	1 (8.3)	6 (25.0)
Ring finger	2 (16.7)	6 (25.0)
Middle finger	2 (16.7)	4 (16.7)
Index finger	0 (0.0)	1 (4.2)
**PIP joints, n (%)**	**7 (58.3)**	**7 (29.1)**
Low baseline severity (≤40°)	3 (25.0)	5 (20.8)
High baseline severity (>40°)	4 (33.3)	2 (8.3)
Little finger	1 (8.3)	5 (20.8)
Ring finger	3 (25.0)	0 (0.0)
Middle finger	0 (0.0)	1 (4.2)
Index finger	3 (25.0)	1 (4.2)

MP = metacarpophalangeal; PIP = proximal interphalangeal.

### Efficacy

**Figure 2:**
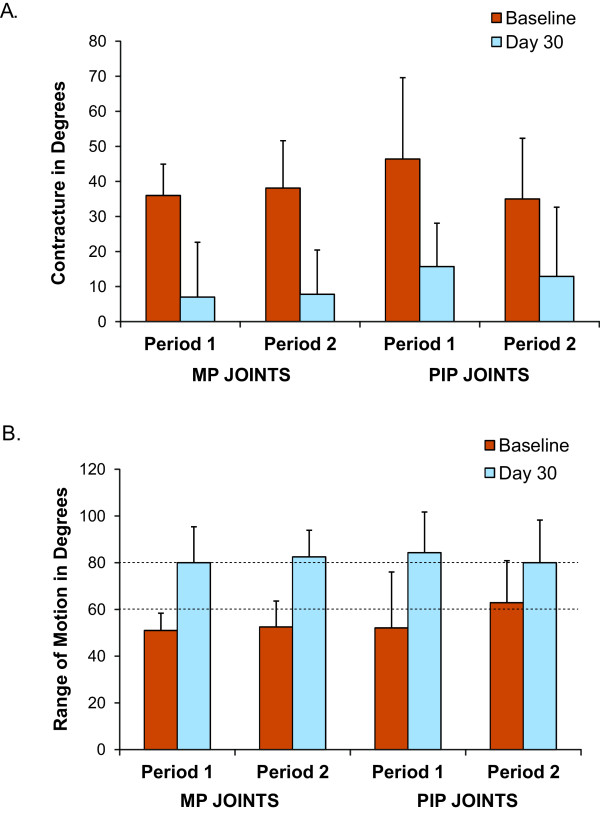
**A. Fixed flexion contracture at baseline and day 30 following a single injection (Period 1) or multiple injections in different joints (Period 2). B.** Range of motion at baseline and day 30 following a single injection (Period 1) or multiple injections in different joints (Period 2). MP = metacarpophalangeal; PIP = proximal interphalangeal.

Results for efficacy endpoints collected following treatment periods 1 and 2 are presented in Table 3. For treatment period 1, the mean reduction in contracture was 29.0 ± 20.7 degrees in the MP joints and 30.7 ± 21.1 degrees in the PIP joints (Figure 2A). This corresponded to a percent change from baseline of 76.7% for the MP joints and 65.3% for the PIP joints. The mean change in range of motion increased by 29.0 ± 21.0 degrees in the MP joints, and 32.1 ± 24.3 degrees in the PIP joints for treatment period 1 (Figure 2B).

**Table 3:** Treatment outcomes

**Study endpoints**	**Period 1 (Day 30)**	**Period 2 (Day 60)**
**MP joints**		
Mean ± SD change in contracture from baseline (degrees)	29.0 ± 20.7	30.3 ± 10.9
Mean ± SD percent change in contracture from baseline	76.7 ± 52.2	83.1 ± 25.6
Mean ± SD change in range of motion from baseline (degrees)	29.0 ± 21.0	30.0 ± 10.8
**PIP joints**		
Mean ± SD change in contracture from baseline (degrees)	30.7 ± 21.1	22.1 ± 4.9
Mean ± SD percent change in contracture from baseline	65.3 ± 32.5	74.3 ± 27.7
Mean ± SD change in range of motion from baseline (degrees)	32.1 ± 24.3	17.1 ± 2.7

MP = metacarpophalangeal; PIP = proximal interphalangeal.

Following multiple injections in different joints given in treatment period 2, the mean reduction in contracture from baseline was 30.3 ± 10.9 degrees in the MP joints, and 22.1 ± 4.9 degrees in the PIP joints (Figure 2A). This corresponded to a percent change from baseline of 83.1% for the MP joints and 74.3% for the PIP joints. The mean change in range of motion increased by 30.0 ± 10.8 degrees in the MP joints, and 17.1 ± 2.7 degrees in the PIP joints (Figure 2B).

Following a single injection in treatment period 1, 9 patients were “very satisfied” and 3 patients were “quite satisfied” with their treatment. All 12 patients were “very satisfied” with their treatment following the multiple injections given concurrently in treatment period 2.

### Pharmacokinetics

All pharmacokinetic samples were below the level of quantification for both AUX-I and AUX-II, indicating no detectable systemic exposure (limit of quantitation for AUX-I = 5 ng/mL; AUX-II = 25 ng/mL).

### Safety

The most common treatment-related AEs that occurred during the study (single- and multiple-dose injection periods) were all local events and included some degree of edema peripheral [swelling in the treated extremity] (100%), contusion [bruising] (100%), pain in treated extremity (100%), lymphadenopathy (83.3%), and pruritus (83.3%). Three adverse events, including edema peripheral, contusion, and pain in the treated extremity, were experienced by the majority of subjects (>80%) in both treatment periods. When comparing treatment-related AEs between treatment periods 1 and 2, a higher incidence of pruritus was observed in treatment period 2 (Table 4). One occurrence of paresthesia was noted in treatment period 1 but was not observed in treatment period 2. Two occurrences of blood blisters and one occurrence of skin discoloration were observed in treatment period 2 but not in period 1. Most of the treatment-related AEs were observed beginning on the day of injection and only a few were observed beginning beyond day 7 after the injection; all resolved by the end of study (with the exception of 1 ongoing incidence of contusion [bruising]), and the mean duration was 2 to 3 weeks for most AEs within both periods. No systemic complications were observed.

**Table 4:** Treatment-related adverse events occurring in all subjects

**Adverse event**	**Period 1 (n = 12)**	**Period 2 (n = 12)**
Patients with ≥1 treatment-related adverse event, n (%)	12 (100)	12 (100)
Blood and lymphatic system disorders		
Lymphadenopathy	6 (50)	7 (58.3)
General disorders and administration site conditions		
Injection site discomfort	0 (0.0)	1 (8.3)
Injection site pain	3 (25.0)	5 (41.7)
Edema peripheral	12 (100)	12 (100)
Injury, poisoning and procedural complications		
Contusion	12 (100)	12 (100)
Skin laceration	2 (16.7)	2 (16.7)
Musculoskeletal and connective tissue disorders		
Pain in extremity	11 (91.7)	11 (91.7)
Nervous system disorders		
Paresthesia	1 (8.3)	0 (0.0)
Skin and subcutaneous tissue disorders		
Blood blister	0 (0.0)	2 (16.7)
Pruritus	5 (41.7)	10 (83.3)
Skin discoloration	0 (0.0)	1 (8.3)

The 5 most common local treatment-related effects were graded according to a 6-point investigator-graded severity scale (Table 5). In general, the differences in severity were not clinically meaningful between treatment periods, and no major differences were observed between treatment periods by 30 days post-injection. Following treatment period 2, all local events resolved by 60 days following injection (eg, had a severity evaluation equal to “none”). The only exception was the above-mentioned ongoing contusion occurring after treatment period 2 that had a severity evaluation equal to “negligible” at day 90.

**Table 5:** Patients with “evident” and “considerable” local effects following 1 or 2 injections for Dupuytren’s contracture

		**Number of Patients (%)**					
**Local Treatment Effect**	**Maximum Severity**^**a**^	**PERIOD 1 (1 CCH injection) (n = 12)**			**PERIOD 2 (2 CCH injections) (n = 12)**		
		**Day 2**	**Day 7**	**Day 30**	**Day 2**	**Day 7**	**Day 30**
Bruising	Evident	9 (75)	2 (17)	0	6 (50)	3 (25)	0
Considerable		1 (8)	0	0	3 (25)	1 (8)	0
Swelling	Evident	2 (17)	1 (8)	0	6 (50)	3 (25)	0
Considerable		5 (42)	0	0	2 (17)	0	0
Pain	Evident	5 (42)	0	0	3 (25)	0	0
Considerable		0	0	0	2 (17)	0	0
Lymphadenopathy	Evident	3 (25)	0	0	1 (8)	0	0
Considerable		0	0	0	1 (8)	0	0
Pruritus	Evident	3 (25)	0	0	0	0	0
Considerable		0	0	0	2 (17)	0	0

CCH = collagenase clostridium histolyticum.

## Discussion

Surgical correction of affected joints is often used to correct multiple contractures on the same hand during the same procedure. However, surgery involving 3 or more fingers, which most likely involves a more extensive operation, has been shown to be significantly associated with increased complications [15]. Typically, complications associated with surgery may include digital nerve or artery injuries, wound-healing complications, dysesthesia/paresthesia, infection, and hematoma [10]. Needle aponeurotomy is another treatment option for patients with DC that is less invasive than open surgery and has been shown to provide beneficial short-term results [16,17]. However, recurrence rates with needle aponeurotomy are higher than those observed with fasciectomy, and many of the same complications associated with fasciectomy have been observed with this technique [16-18].

CCH is currently approved in both the US and EU for adults with DC for treatment of a single palpable cord during any 30-day treatment period, and has become an acceptable alternative to surgical correction of DC. The results from this exploratory study suggest comparable improvement in contracture and range of motion per injection of CCH when given as a single injection or when multiple injections are administered concurrently, although this study was not powered for statistical significance. For the MP joints, mean change in contracture was 29.0 ± 20.7 degrees following single injection or 30.3 ± 10.9 degrees following a single injection in the multiple injection phase. Similar results were observed in the change in range of motion with increases of 29.0 ± 21.0 and 30.0 ± 10.8 degrees in MP joints for single and multiple phase injections, respectively. Comparable but slightly less improved results were observed for PIP joints following the single or multiple injection phase, with mean change in contracture of 30.7 ± 21.1 vs 22.1 ± 4.9 degrees and change in range of motion of 32.1 ± 24.3 vs 17.1 ± 2.7 degrees, respectively.

In previously published studies that evaluated CCH treatment of a single cord treated sequentially, the mean reduction in contractures observed for MP joints (40.8 and 42.0 degrees) and PIP joints (32.7 and 32.2 degrees) were comparable to mean reduction in contractures observed following the single and multiple injection phase in this study [13,14,19]. Comparable results were observed for changes in range of motion in MP joints for the single (29.0 ± 21.0 degrees) and multiple injection phase (30.0 ± 10.8 degrees) and PIP joints following the single injection phase (32.1 ± 24.3 degrees) from this study when compared to 40.6 and 40.0 degrees for MP joints and 29.0 and 32.0 degrees for PIP joints reported in previously published studies [13,14]. Following the multiple injection phase in which a PIP was one of the joints injected, data values for mean change in contracture and change in range of motion were slightly lower in this study compared to those reported previously [13,14]. Some of these differences may be due in part to the fact that the PIP joints did have a higher baseline range of motion. Additional factors that may account for these differences may include the small number of subjects in this study and the variability in treatment response often observed among individuals.

In this study, all blood samples were below quantifiable levels for both AUX-I and AUX-II for both treatment periods, consistent with results from an unpublished single-dose pharmacokinetic study [19]. The results from this study suggest that single and multiple concurrent injections are similarly well tolerated. As expected, a slightly greater severity of treatment-related AEs was observed during treatment period 2, however, these AEs resolved by day 30 after each injection. These local events were directly solicited from investigators in an open label setting, which may impact the reporting rate. No serious treatment-related AEs were observed in this study following both single and multiple injections. Reported AEs were as expected following single and multiple injections and compared to previously reported data. Common AEs reported in this study and in previously published studies were local reactions to CCH injection, including edema peripheral, contusion, pain in extremity, injection site pain, injection site hemorrhage, and lymphadenopathy [13,14]. No reports of nerve or arterial injury were observed following CCH injection in this or the previously published studies [13,14]. Overall, the safety results from this study are consistent with previously published data and indicate that increasing the number of injections given concurrently does not meaningfully increase the rate or severity of observed AEs.

## Conclusions

The results from this study suggest that two cords can be treated concurrently with CCH with comparable efficacy and safety as when a single cord is treated. The ability to give multiple injections, without the need to wait 30 days between treatments, would allow for a more rapid and effective treatment of multiple affected joints in a fashion similar to the surgical paradigm, and constitute a significant advantage for both patient and physician.

## Competing interests

SC received research support from Auxilium Pharmaceuticals and owns Auxilium stock. DG is an investigator for Auxilium Pharmaceuticals and owns Auxilium stock. JT, GK, NJ, and BC are employees of Auxilium Pharmaceuticals, who provided funding for this clinical study.

## Authors’ contributions

SC and DG served as principal investigators of this study and were involved in study design, oversaw the conduct of the trial, and were involved in data collection and interpretation. JT, GK, NJ, and BC contributed to study design and were involved in data interpretation. Statistical analyses were performed by BC. All authors contributed to the writing, reviewing and editing of the manuscript, and approved the final draft.

## Pre-publication history

The pre-publication history for this paper can be accessed here:

http://www.biomedcentral.com/1471-2474/13/61/prepub
